# Morality and Values in Sports Among Young Athletes: The Role of Sport Type and Parenting Styles – A Pilot Study

**DOI:** 10.3389/fpsyg.2021.618507

**Published:** 2021-02-10

**Authors:** Yosi Yaffe, Orr Levental, Dalit Lev Arey, Assaf Lev

**Affiliations:** ^1^Department of Education, Tel-Hai Academic College, Qiryat Shemona, Israel; ^2^Department of Phsyical Education, Tel-Hai Academic College, Qiryat Shemona, Israel; ^3^Department of Psycology, Tel-Aviv-Yaffo Academic College, Tel Aviv-Yaffo, Israel; ^4^Department of Sports Therapy, Ono Academic College, Kiryat Ono, Israel

**Keywords:** parenting style, morality and values, Israel, adolescents, athleths

## Abstract

Given the great importance of morality and values in modern sports, especially among young athletes, in this pilot study, we sought to broaden the exploration of the factors that may play role in these contexts, which have not been widely researched to date. Accordingly, the study tested the relationships between sport type (team or individual) and parenting styles (authoritative vs. non-authoritative), and moral decision-making in sport and sport values among 110 adolescent athletes whose age ranges from 11 to 22 (*M* = 16.04, *SD* = 2.86). The findings indicated that participants with authoritative parents, as compared to those with non-authoritative parents, are significantly less accepting of cheating in sport, while they also tend more to keep winning in proportion and hold significantly stronger moral values toward sports. Moreover, participants whose main sport is a team sport type tend to accept more cheating and gamesmanship than participants whose main sport is an individualistic sport type. While no differences were recorded between these groups in moral values, team athletes tend to value status in sport more than individual athletes, while the latter tend to value competence regarding their sport. The implications of the findings are discussed in light of no interaction between the effects of parenting styles and sport type on moral and sport values.

## Introduction

The professionalization and commercialization of sports emphasize the need for wins over participation. This combination of sportsmanship and competitiveness has created a conflict between values and functionality (Levental, 2020). Thus, a moral dilemma arises, related to the desire and need to win against the importance of participation and following the rules of the game, or as Jones and David Howe (2005) suggest, a contest between fairness and merit (Jones and David Howe, 2005). According to the social learning theory, behavioral change is a product of reinforcement and punishment along with behavioral imitation of other meaningful individuals. Thus, there is importance in examining the internalization of moral standards among young athletes (Whitehead et al., 2013). Furthermore, the distinction made between the competition-game context and life itself should also be addressed. According to Shields and Bredemeier (1994, 2007), the rules of some competitions often allow for greater egocentrism and moral flexibility than life outside of sports. That is, the environmental context influences ethical attitudes and behavior. Ethical perceptions among young athletes should, therefore, be examined in two aspects. The first is identifying values and their behavioral expressions (see Kavussanu, 2008), and the second is the effects of different variables, personal and environmental, on the formation of these perceptions. For example, the presence of other individuals or rewards and penalties (Lee and Cockmen, 2013). One aspect related to both environmental influences and behavior is the culture in which the sport is performed. A study by Whitehead and Goncalves (2013) showed that different sports values can be found in different countries. Demographic aspects may also have an impact on adopted values. More than half a century ago, Webb (1969) found that increasing age indicates a higher commitment to victory than fair play. In addition, with increasing age, there is a wider acceptance of the use of aggressive behavior during sports (Lee and Williams, 1989). The age factor was also examined in an article by Lee et al. (2013) that found that values associated with sport become less significant with increasing age. Another study by Lee et al. (2013) found that older athletes (14–16) more than young athletes (11–13) reported higher Acceptance of Cheating as well as Acceptance of Gamesmanship.

The present article focuses mainly on the implications of the type of sport – individual or team – on the ethical attitudes of young athletes. This is based on the understanding that structural aspects in both types of sport affect the athletes’ environment and consequently their ethical perception. One example of the difference between athletes from a team sport vs. an individual sport can be found in the study of Boardley et al. (2015) about athletes’ perceptions regarding the use of performance-enhancing drugs. They found that due to the team sharing responsibility for winning, athletes who are part of a team develop a more lenient approach regarding the possible benefit of using drugs to gain a competitive advantage. This is due to the fact that the illegal advantage gained by one athlete has a relatively marginal effect on the result of the game. This comparative aspect was also reflected in a study by Woolf and Mazanov (2017). However, while a difference was found concerning doping between athletes at different competitive levels, no differences were found in the perceptions of athletes from individual and team sports. Also, another study found no differences in terms of ranking sports values among young athletes from individual and team sports (Lee et al., 2013). Whereas regarding ethical decisions in youth sport, it was found that there are differences between athletes from team sports and individual sports in two out of three categories (Lee et al., 2013). Young athletes participating in team sports received a higher score on Acceptance of Cheating and Acceptance of Gamesmanship but were no different from individual sport athletes in Keeping Winning in Proportion. The difference between team and individual sports in terms of ethical perception may stem from the different approaches of coaches in different fields and emphasizing personal development vs. sporting success (Peláez et al., 2016). The moral reasoning of athletes in a team is greatly influenced both by its moral climate (Kavussanu et al., 2002) and by the orientation of team performance vs. mastery, which directs the behavioral norms of individuals (Ommundsen et al., 2003). Naturally, a major part of the moral perception of the sport depends on rivalry. Therefore, a moral-ethical difference could be the result of different perceptions of the individual opponent or rival team. Vallerand et al. (1997) found that team athletes tend to express less concern about their opponent than athletes in an individual sport. Moreover, the findings of Calmeiro et al. (2015) show that athletes from team sports exhibit moral reasoning at lower levels than athletes from individual sports. One of the explanations offered by the authors is that in team sports, due to the direct encounter with an opponent, self-improvement contributes to victory as well as impairing the opponent’s abilities. Another explanation for this may be the collective moral responsibility and the reduction of the role of the individual athlete within the team (Rudd and Stoll, 2004; Boardley and Kavussanu, 2009).

When discussing young athletes’ morality and values system in sport, the youngsters’ individual differences in fundamental morality should also be considered. A central factor affecting children’s and adolescents’ development of morality is the family environment, with parents specifically playing the most important role in this issue (Smetana, 2015). Through socialization processes, parents impart the habits, values, and norms congruent with adaptation to their culture (Baumrind, 1980) which shape and construct their moral knowledge. As the primary caregivers and raisers who spend most time with the child and closest to them, parents normally serve as the main socialization agents whose role in the child’s moral development is crucial. Parents are also central in the context of moral development due to their affective relationship with their children and their responsibility to educate and discipline them while instilling moral values (Wainryb and Recchia, 2014; Smetana, 2015). They convey moral values *via* both cognitive and affective components (Smetana, 1999) that underlie the family’s social interactions and parent-child relationships. In the context of sports, the role of socialization is key to understanding aspects of an athlete’s sensations, ideals, and values (see: Lev, 2019). Regarding family and the process through which young athletes learn the values and norms of sports culture, children’s experiences can be molded based on their exposure to motivational, cognitive, and affective responses (Harwood et al., 2019). Family influences also affect young athletes’ embrace of positive values and satisfaction, and their approach and comportment, in times of stress, toward sport and physical activity (Tamminen et al., 2016; Lev et al., 2020). In this regard, a recent study (Danioni et al., 2017) showed that adolescent athletes whose parents endorsed core values in sport gave great importance to competence values (e.g., skillsets and accomplishments) and moral values (e.g., living up to obligations and fairness), but attributed little importance to status values (e.g., titles and winning). In other words, adolescent athletes valued sportsmanship above gamesmanship. However, families and parents vary in a manner in which they socialize their children, which, according to a large body of research, may be related to various differences in children’s moral, behavioral, and emotional development (Pinquart, 2017a,b; Pinquart and Gerke, 2019; Fatima et al., 2020). While both parents play a significant role in their child’s sporting activity, there are differences in the roles that the genders play in conveying values and norms through the socialization of children regarding sports (Palomo-Nieto et al., 2011). In this context, socialization theorists and researchers have focused on different types of parental behaviors, practices, and styles within the family that influence the moral internalization in children, and which are assumed to subsequently effect the development of the children’s broader personality and emotional characteristics. Parenting is a broad construct that comprises stable and durable attitudes and behaviors toward child-rearing (Smetana, 2017), while the theoretically widely-used terminology in the literature to describe its substance and types is parenting styles (Baumrind, 1966). The concept of parenting styles refers to certain types of parenting that are characterized by distinct attitudes and behaviors toward the child and constitute a form of marital climate (Yaffe, 2020). Three discerned styles of parenting are known in the literature as authoritative, indulgent (or permissive), and authoritarian (Maccoby and Martin, 1983; Baumrind, 1991), which generally differ by the practices they use and the type of control they exert in raising their children. Authoritative parents consistently incorporate behavioral control with providing warmth and emotional support and closeness (Baumrind, 1966; Maccoby and Martin, 1983). They establish their offspring’s socialization on reasoning, negotiation, and shared decision-making, while setting consistent limits and rules and encouraging autonomy (Baumrind, 1966, 1968, 2005; Yaffe, 2020). Authoritarian parents display a discerned type of parental authority, as they exert strict and intrusive control over the child, while avoiding negotiation, using punishment, and maintaining an emotional distance. Finally, permissive parents tend to practice lax control, to avoid punishing, and to maintain emotional closeness (Baumrind, 1966, 1968, 2005).

Indeed, as compared to an offspring from non-authoritative families, adolescents who were raised by authoritative parents exhibit several developmental socio-emotional advantages: they academically outperform their counterparts at school (Spera, 2005; Pinquart and Kauser, 2018), manifest better psychological adjustment in terms of lower depression and anxiety (Pinquart, 2017a; Yaffe, 2018a), tend to be more morally developed while engaging with less behavior and externalized problems (Freeze et al., 2014; Pinquart, 2017b; Fatima et al., 2020), have higher self-esteem (Pinquart and Gerke, 2019; more), and are more likely to be health behaviorally oriented (Vollmer and Mobley, 2013; Yaffe, 2018b). Accordingly, Darling and Steinberg (1993) submitted that since offspring of authoritative parents tend to endorse their parents’ parental authority and feel obligated to obey their rules, they are more convenient for parental socialization and, consequently, tend to internalize more intensively their parents’ social and moral values.

### The Current Study

This research is a pilot study aiming to explore the parenting and sport factors associated with young athletes’ moral values in sports. In light of the inconsistencies reflected from the body of research on the moral-ethical values of young athletes from different sport types, the current study sought to broaden that scope by also accounting for the youngsters’ familial background as an additional factor explaining their ethical values in sport. As demonstrated by the above literature review, parental socialization of children and adolescents plays a substantial role in their moral development, which could also specifically apply to young athletes’ moral values in the context of sport. Yet, the research in the area of morality and values in sports among young athletes has focused on sport variables and has failed to consider adequately the role of interpersonal differences in familial background, such as parenting styles. In this respect, incorporating determinants of general morality (i.e., parenting) with those of sport morality (i.e., sport type) could be promising in advancing our understanding regarding athletes’ moral and values system in sport, which becomes crucial with the growing popularity of sports in the modern era. In this regard, the following hypotheses are tested in the current study:

- Young individual-sports athletes will differ from young team-sports athletes in moral decision-making in sport and sport values, so that the former would express lower acceptance of cheating and gamesmanship, a stronger tendency to keep winning in proportion, and stronger moral values in sport.
- Young athletes who perceive their parents as authoritative will differ from young athletes who perceive their parents as either authoritarian or permissive in moral decision-making in sport and sport values, so that the former would express lower acceptance of cheating and gamesmanship, a stronger tendency to keep winning in proportion, and stronger moral values in sport.

## Materials and Methods

### Participants and Procedure

The sample included 110 adolescent athletes (97 males and 13 females) whose age ranges from 11 to 22 (*M* = 16.04, SD = 2.86), with the majority of them Jewish students (84.5%) and the rest Arab students. They belonged to families whose sizes ranged from 2 to 10, with a mean size of 4.95 ± 1.25. The participants were all engaged in regular competitive and professional sports activities, which included cycling, aerobic sports, motor sports, tennis, soccer, basketball, fencing, gymnastics, triathlon, climbing, and swimming. As part of our research purposes, these sports were classified into two groups of sport type: individual sports and team sports. Of the sample of students, approximately 24% reported having some diagnosed learning disability. Since their scores on the dependent variables (i.e., the moral decision-making and system values in sport) did not statistically differ from those of the sample of students without learning disabilities, the former were equally included in the general sample as part of the analyses of the research hypotheses (apart from this reason, separate analyses for this group could not be applied due to its small sample size). The data collection procedure was carried out using a snowball sampling method, where the participants were recruited due to their regular engagement in professional sport *via* public and personal announcements to take part in the current pilot study. Participants (from organized sport groups or individually) were initially informed about a pilot study dealing with ethical perceptions in sport and were asked whether they would be willing to take part in completing anonymous questionnaires. Those students who expressed their interest were given an online link where they could read a detailed explanation about the study’s objectives and the participation terms. Filling out the research forms was conditioned on signing an online informed consent in advance (for participants ages over 18) and, for minor participants, in giving their assent and obtaining parental permission for taking part in the study. An *ad-hoc* institutional ethics committee reviewed and verified the data collection program prior to applying its procedure.

### Measures

#### Parental Authority Questionnaire

The Parental Authority Questionnaire (PAQ; Buri, 1991) contains 30 items and is used to classify parents into one of Baumrind’s three parenting styles (Baumrind, 1971), based on the adolescent’s self-report: *Authoritative* (10 items, e.g., “As I was growing up, once family policy had been established, my parents discussed the reasoning behind the policy with the children in the family”), *Authoritarian* (10 items, e.g., “As I was growing up my parents did not allow me to question any decision they had made”), and *Permissive* (10 items, e.g., “As I was growing up my parents seldom gave me expectations and guidelines for my behavior”). The response scales for an item range from 1 (strongly disagree) to 5 (strongly agree). The index for each parenting style is the sum of the relevant items of each scale. Thus, the total score for each parenting scale ranges from 10 to 50, with a higher score reflecting a higher specification of the style. PAQ is a valid questionnaire with relatively high internal consistency and test-retest reliabilities (0.74–0.78; see: Buri, 1991; Smetana, 1995), widely used in Israel (e.g., Enten and Golan, 2009; Yaffe, 2018a) and around the world to measure Baumrind’s (1971) three basic styles of parenting. It was originally developed and validated in English using high-school and college students (Buri, 1991), and has previously shown supporting evidence of validity and reliability in its Hebrew version with early adolescents (Yaffe, 2018a). The current study recorded alpha coefficients for the permissive, authoritarian, and authoritative scales of 0.64, 0.88, and 0.79 (respectively). The scores obtained in the current sample for the instrument’s scales appear in Table 1.

**Table 1:** Descriptive statistics and correlation matrix for the research variables.

S. No.	Variable	1	2	3	4	5	6	7	8	9
**Parenting styles**										
1.	Permissive	-								
2.	Authoritarian	−42^***^	-							
3.	Authoritative	0.38^***^	−0.42^***^	-						
**Moral decision-making in sport**										
4.	Acceptance of cheating	0.05	0.26^***^	−0.31^***^	-					
5.	Acceptance of gamesmanship	−0.01	0.17	−0.15	0.48^***^	-				
6.	Keeping winning in proportion	0.01	0.03	0.21^*^	−0.22^*^	−0.03	-			
**Sport values**										
7.	Moral values	0.05	−0.14	0.30^**^	−0.56^***^	−0.40^***^	0.50^***^	-		
8.	Competence values	0.15	−0.17	0.11	−0.18	−0.08	0.25^**^	0.53^***^	-	
9.	Status values	−0.03	0.22^*^	−0.25^**^	0.33^***^	0.41^***^	0.04	0.03	0.41^***^	-
	Mean	27.48	26.93	35.0	2.06	2.79	4.20	3.50	4.02	2.90
	SD	5.42	8.08	6.48	0.90	1.04	0.74	0.87	0.76	1.06

* *p* ≤0.05.

** *p* ≤ 0.01.

*** *p* ≤ 0.001.

#### Attitudes to Moral Decision-Making in Youth Sport Questionnaire

This is 9-item questionnaire with three scales designed to measure attitudes toward *Acceptance of Gamesmanship* (e.g., “Sometimes I waste time to unsettle the opposition”), Acceptance of Cheating (e.g., “I would cheat if I thought it would help me win”), and *Keeping Winning in Proportion* (e.g., “It’s OK to lose sometimes because in life you do not win everything”). In the current study, we recorded Cronbach’s alpha values of 0.80, 0.83, and 0.72 for these three scales, respectively, which are adequate reliability indexes of internal consistency given the small number of items included in the scales. Each scale comprises three items whose responses are given on a 1–5 Likert response scale. Responses should be averaged to produce the scales’ *mean score*. The Attitudes to Moral Decision-Making in Youth Sport Questionnaire (AMDYSQ) was concurrently validated against several external variables (such as commitment to sport, respect for conventions, respect for opponents, and respect for rules) and its measurement demonstrated gender invariance (Lee et al., 2013). The AMDYSQ’s subscales have also been shown to have negligible associations with social desirability scale. Normally, the *Acceptance of Gamesmanship* and *Acceptance of Cheating* scales correlate positively, while each of these scales correlates negatively with the *Keeping Winning in Proportion* scale (Lee et al., 2007).

#### Youth Sport Values Questionnaire

This 13-item questionnaire is a modified version of the original Youth Sport Values Questionnaire (YSVQ; (Lee et al., 2000 2008), designed to measure young athletes’ values system in sports. Its three subscales contain higher-order measure *moral values* (five items; e.g., “I try to be fair”), *competence values* (four items; e.g., “I improve my performance”), and *status values* (four items; e.g., “I win or beat others”; Lee et al., 2013). The mean scores are obtained by averaging the item scores for each scale. Normally, *Competence* values correlate more highly with *moral* values than with *status* values and the correlation between *moral* and *status* values is lowest of all (Lee et al., 2013). The English YSVQ-2 was translated and used in studies across, at least, eight different countries outside the United Kingdom, which their findings underpinning the suitability of the questionnaire for different cultural contexts. The YSVQ-2’s subscales have been shown in previous studies to relate logically to the AMDYSQ’s subscales, especially the moral and competence values, which were negatively correlated with the acceptance of cheating and gamesmanship (see Whitehead et al., 2013). The developers reported a good 4-week test-retest reliability indication for the questionnaire’s scales. In the current study, we obtained acceptable indices for the scales’ internal consistency reliability (that is, the *moral* values, the *status* values, and the *status* values scales), with their Cronbach’s alpha coefficients ranging between 0.71 and 0.80. For the purposes of the current study, the authors adapted and translated the English forms of the AMDYSQ and the YSVQ-2 using the back-forward translation method. The scores obtained for these scales in the current study are presented in Table 1.

Both sport questionnaires (i.e., the AMDYSQ and the YSVQ-2) were developed with the 12–16 years age group of young athletes, implying that their content could fit close and older age populations.

### Ethics

The study involving human participants (as described above) was reviewed and approved by an institutional ethics committee of Ohalo Academic College, Israel.

## Results

At first, we tested the scales’ scores and the zero-order correlations between the study variables, to identify the general tendencies of the results. The study’s operational variables included the parenting styles scales, the moral decision-making, and the sport values scales, which each contains three subscales. As appears in Table 1, the parenting scales are significantly intercorrelated in accordance with the expected directions, with the authoritative and permissive scales being reversely associated with the authoritarian scale. The parenting scales also exhibited numerous significant connections with the moral decision-making and sport values subscales, as the authoritative parenting was negatively correlated with acceptance of cheating and status values and positively correlated with moral values and keeping winning in proportion. Relative to the authoritative scale, the authoritarian scale was inversely correlated with most of the moral decision-making and sport values subscales. That is to say that young athletes who perceive their parents as more authoritative (rather than authoritarian) are more likely to embrace moral values in sport, to keep winning in proportion, and to reject cheating.

We also observed some significant correlations between the moral decision-making and the sport values subscales, which partially reflect the correspondence and agreement between the measurements. Hence, acceptance of cheating was considerably correlated with the accepting of gamesmanship, while these two variables were simultaneously associated with lower levels of moral values and higher levels of status values in sport. And, while the sample of athletes who hold high moral values less favored cheating and gamesmanship, they did value competence in their sport and tended more to keep winning in proportion.

### Testing the Research Hypotheses

We generally hypothesized that young athletes’ morality and values in sport would differ by their perceptions of their parenting styles and their main sport type. As mentioned, the dependent variables were measured in the current study *via* the youth “moral decision making” in sport and the “youth sport values” scales. Parenting styles were classified into groups using the highest score among the three continuous scales (i.e., permissive, authoritarian, and authoritative). This parenting categorization resulted in considerably unbalanced group sizes, with the vast majority classified as authoritative, forcing us to use only two groups of parenting (that is, authoritative and non-authoritative, which includes permissive and authoritarian parenting). Although parents who use demandingness (that is, authoritarian) and parents who do not use demandingness (that is, permissive) are essentially different in their styles, classifying them as non-authoritative parents is theoretically logical and has been operationally utilized in previous studies for various comparison-based purposes against authoritative parents (Steinberg et al., 1991; Zuquetto et al., 2019). To test the research hypotheses, we conducted a multivariate ANOVA (MANOVA) for the differences in these scales’ scores by parenting style and sport type (displayed in Table 2). Since the study’s demographics had merely negligible effects (for the most part not statistically significant) on the dependent variables, they were not taken into account as part of the analysis.

**Table 2:** Means, SDs, and the results of multivariate ANOVA (MANOVA) analysis for the differences between youth’s moral decision making in sport and sport values by parenting style and sport type.

	Parenting style				Sport			
	Authoritative (*n* = 67)	Non-authoritative (*n* = 43)	*F* (1,106)	Partial *η^2^*	Team (*n* = 65)	Individual (*n* = 45)	*F* (1,106)	Partial *η^2^*
**Moral decision-making**								
Cheating	1.82 (0.79)	2.44 (0.94)	13.07^***^	0.110	2.27 (0.88)	1.76 (0.85)	6.79^**^	0.060
Gamesmanship	2.70 (1.10)	2.93 (0.93)	0.83	0.001	3.14 (0.94)	2.28 (0.97)	17.64^***^	0.143
Proportion	4.29 (0.72)	4.05 (0.73)	4.28^*^	0.040	4.26 (0.65)	4.11 (0.84)	2.19	0.020
**Sport values**								
Moral values	3.69 (0.82)	3.20 (0.88)	9.78^**^	0.084	3.45 (0.69)	3.58 (1.09)	0.03	0.000
Competence values	4.09 (0.75)	3.91 (0.77)	0.51	0.001	3.89 (0.63)	4.19 (0.88)	5.02^*^	0.045
Status values	2.74 (1.15)	3.14 (0.85)	2.85	0.026	3.08 (0.94)	2.62 (1.06)	4.41^*^	0.040

Partial *η^2^* is a measure of effect size.

* *p* ≤ 0.05.

** *p* ≤ 0.01.

*** *p* ≤ 0.001.

The multivariate tests indicated significant general effects of parenting styles (Wilks’ lambda = 0.84, *p* < 0.01) and sport type (Wilks’ lambda = 0.69, *p* < 0.001) on the dependent variables. An inspection of the individual effects using the between-subjects tests (Table 2) revealed a significant main effect of parenting styles on the cheating, proportion, and the moral values subscales, with the largest effect size observed on cheating. This indicates that participants with authoritative parents, as compared to those with non-authoritative parents, are significantly less accepting of cheating in sport, while they also tend more to keep winning in proportion. These participants (i.e., youngster athletes with authoritative parents) also hold significantly stronger moral values toward sports, but they do not differ from their counterparts (i.e., youngster athletes with non-authoritative parents) in the acceptance of gamesmanship and in competence and status values.

Furthermore, we recorded main effects for the sport type on the subscales of cheating, gamesmanship, competence, and status values, with the largest effect size observed on gamesmanship. This means that participants whose main sport is team sport type tend to accept more cheating and gamesmanship than participants whose main sport is an individualistic sport type. While no differences were recorded between these groups in moral values, they did significantly differ in their values of competence and of status. Thus, team athletes tend to value status in sport more than individual athletes, while the latter tend to value more competence regarding their sport. These differences in sport values by sport type were of the smallest size as compared to the other effects observed here, yet they were statistically significant. Finally, whereas both parenting styles and sport type affected several variables of moral decision-making and the sport values simultaneously, their effects in either of these contexts did not interact. This generally denotes that differences between the sample of participants with authoritative parents and those with non-authoritative parents in moral decision-making and sport values apply similarly for both team athletes and individual athletes (and vice versa).

## Discussion

The study sought to examine the relationships between young athletes’ sport type and parenting styles and their moral and values system in sport, in an attempt to determine whether and how young athletes vary in their moral decision making in sport and sport values by the type of sport they specialize in (i.e., individual vs. team sport) and by their parents’ background (i.e., authoritative vs. non-authoritative parenting style). The first research hypothesis held that young athletes engaged in individual sports express lower acceptance of cheating and gamesmanship, a stronger tendency to keep winning in proportion, and stronger moral values in sport. The findings of the study show that there is indeed a significant difference between them and athletes from team sports. These findings are consistent with those of Boardley et al. (2015), according to which group effort overshadows the individual and, therefore, forms of cheating, such as taking drugs, do not greatly impact the result. Similar results were also found by Lee et al. (2013), who found that athletes in individual sports received lower scores on Acceptance of Cheating and Acceptance of Gamesmanship. However, in contrast to the current study, no difference was found regarding Keeping Winning in Proportion. A possible reason for this is that cultural differences in moral standards in different societies (Whitehead and Goncalves, 2013). In contrast to the attitude toward cheating, which relies on shared responsibilities, the importance of winning is a product of social perceptions. Thus, local norms of competitive behavior shape the perceptions of young athletes. Furthermore, the competitive level may have an impact, as found in the studies of Woolf and Mazanov (2017). According to their study, the differences in ethical perceptions between the team and individual athletes decrease when the level of play is non-competitive. This is partially similar to the sports in which the current study’s participants take part.

The findings of the present study show no differences between the two groups in their moral values. These findings are consistent with previous studies, such as those of Lee et al. (2013). As previously argued, earlier studies found that athletes from team sports exhibit moral reasoning at lower levels than athletes from individual sports. This is because team athletes express less concern for their opponent. The proposed rationale is that in a direct encounter, impairment of the opponent’s abilities is tantamount to self-improvement, or alternatively, the collective responsibility that diminishes the influence of the individual in the group (Boardley and Kavussanu, 2009). These aspects were expressed in the fact that the young athletes from the team sports emphasized the importance of status while the athletes in the individual sports preferred competence. An explanation for this can be found in the effect of group climate on moral reasoning (Kavussanu et al., 2002) and by the orientation of team performance (Ommundsen et al., 2003). Because the teams’ goal is collective success, self-improvement is not necessarily measurable and, therefore, receives less attention.

Our second hypothesis dealt with the relationships between young athletes’ parents’ parenting styles and their moral and values system in sport, assuming that offspring who were raised by different types of parents may vary in their moral development and their fundamental morality. The current study’s findings are in line with previous findings suggesting that young athletes raised by parents who communicate high morals and values as part of socialization thus carry those traits into their sport activity (Danioni et al., 2017). Partially consistent with our hypothesis, we found that the sample of youngsters who perceived their parents as authoritative, compared to their peers who perceived their parents as non-authoritative, expressed lower acceptance of cheating and a stronger tendency to keep winning in proportion. Along with the formers’ stronger tendency to hold stronger moral values in sport, our findings generally suggest that young athletes from authoritative families are more morally oriented in sports than those from non-authoritative families. Assuming that these moral tendencies in sport reflect the youngsters’ wider moral value system, this conclusion principally corroborates the premise that adolescents whose parents are authoritative are more prone to parental socialization and, therefore, more intensively internalize and apply their parents’ social and moral values in various areas of their lives (Darling and Steinberg, 1993). Indeed, the current study’s findings are consistent with previous findings suggesting that children and adolescents who were raised by authoritative parents might be more morally developed than those who were raised by non-authoritative parents (Freeze et al., 2014; Pinquart, 2017b; Fatima et al., 2020). Interestingly, the effects of parenting styles on moral and sport values discovered here did not interact with the athletes’ sport type, which means that the differences between athletes from authoritative and non-authoritative families in these variables apply equally to both individual and team sports.

Moreover, alongside other social and emotional differences observed between offspring with a discerned parental background (Spera, 2005; Pinquart, 2017a,b; Pinquart and Kauser, 2018; Yaffe, 2018a), this study initially reveals another unique outcome variable to which parenting styles could be related in adolescence. While studies dealing with the consequences of parent-child relationships on adolescents’ development tend to focus primarily on central psychological, educational, and mental health variables (e.g., academic achievements, anxiety, depression and self-esteem, pro-social behavior, etc.; Steinberg, 2001), the current findings are the first to demonstrate the exclusive link between parenting styles and the outcomes of young athletes’ morality in sport. Apart from the significance these findings may bear in terms of the importance of parenting with relation to child-socialization, they also can have implications for the origins of sport morality among professional athletes. In this context, perhaps our findings imply that athletes’ moral values in sports are rooted in the fabric of their relationships with their parents, whose parenting styles primarily shaped their more general moral thinking. Since the current study design is cross-sectional in nature, a causal conclusion cannot be drawn from its findings regarding the relationship between parenting styles and young athletes’ morality in sport. Therefore, this assumption must be further inspected in studies using a more advanced research design suitable to determine the causal direction in the associations between these variables. Given that most studies about moral and values in sport have focused exclusively on sport variables, broadening the inspection of the origins of morality in sports to family background among young athletes might be promising.

The study findings are limited in several respects. First and foremost, with the sample of athletes composed mainly of men, the study’s conclusions regarding both effects sport type and parenting styles on moral values in sports, could not be generalized equally for women athletes. Further study with more balanced gender-distribution is required to determine how young men and women athletes differ on the basis of the study variables. Also, with a relatively small sample size which is unrepresentative of the young athletes’ population in Israel, the findings of this pilot study should be treated cautiously and predominantly serve to cultivate further topic-related research.

## Data Availability Statement

The raw data supporting the conclusions of this article will be made available by the authors, without undue reservation.

## Ethics Statement

The studies involving human participants were reviewed and approved by the institutional ethics committee of Ohalo Academic College, Israel. Participants provided their written informed consent to participate in the study.

## Author Contributions

DLA and AL contributed to analyzing the data and interpreted the findings. They also expanded the literature review following the reviewers’ comments. DLA and AL, along with OL and YY wrote and approved the final revision of the article.

### Conflict of Interest

The authors declare that the research was conducted in the absence of any commercial or financial relationships that could be construed as a potential conflict of interest.
